# Bird Assemblages in Coffee Agroforestry Systems and Other Human Modified Habitats in Indonesia

**DOI:** 10.3390/biology11020310

**Published:** 2022-02-15

**Authors:** Muhammad Ali Imron, Marco Campera, Dennis Al Bihad, Farah Dini Rachmawati, Febrian Edi Nugroho, Budiadi Budiadi, K. Fajar Wianti, Edi Suprapto, Vincent Nijman, K.A.I. Nekaris

**Affiliations:** 1 Faculty of Forestry, Universitas Gajah Madah, Yogyakarta 55281, Indonesia; dennis.al.b@mail.ugm.ac.id (D.A.B.); farah.dini.r@mail.ugm.ac.id (F.D.R.); febrian.edi.n@mail.ugm.ac.id (F.E.N.); budiadi@ugm.ac.id (B.B.); kfajar@ugm.ac.id (K.F.W.); 2 Sustainable and Resilient Futures Network, Oxford Brookes University, Oxford OX3 0BP, UK; mcampera@brookes.ac.uk (M.C.); vnijman@brookes.ac.uk (V.N.); anekaris@brookes.ac.uk (K.A.I.N.); 3 ARuPA, Yogyakarta 55284, Indonesia; edisuprapto@gmail.com

**Keywords:** shade grown coffee, biodiversity, Java, forest conservation, avian, wildlife-friendly, sustainable

## Abstract

**Simple Summary:**

Given the high degree of deforestation in the tropics due to shifting agriculture, it is a priority for conservation to find sustainable agriculture systems. We assessed bird communities over 1228 plots from 21 sites in the highly populated island of Java, Indonesia. We found that commercial coffee polycultures (i.e., fields comprised of coffee plants, other crops and/or fruit trees, and diverse shade trees) could sustain similar levels of bird abundance, diversity, and richness than coffee systems under natural forests and community managed forests. Commercial coffee polyculture fields host higher bird abundance, diversity, and richness than sun-exposed coffee fields, fields with other crops or fruit trees, and tree farms. We provide evidence that complex commercial agroforestry systems can host similar levels of biodiversity to agroforestry systems under natural forest.

**Abstract:**

Deforestation in the tropics is mainly driven by the need to expand agriculture and forestry land. Tropical cropland has also undergone a process of intensification, particularly evident in regions that are the main exporters of deforestation-driven commodities. Around 25 million people in the world depend on coffee production, which has a profound contribution to global biodiversity loss through agricultural extensification and intensification. Nevertheless, coffee agroforestry systems have been postulated to serve as an alternative refuge for biodiversity across different regions. We aim to compare bird abundance, diversity, and richness in commercial polyculture coffee systems (i.e., the highest degree of habitat complexity that can be achieved in coffee fields after deforestation) with other coffee agroforestry systems and human modified habitats in Java, Indonesia. We collected data in 21 sites (1228 points) on Java from February to August 2021 using the point sampling method. Via generalised additive models, we tested whether the abundance, diversity, and richness of birds were different between different human modified habitats including other potential predictors such as elevation, distance to protected areas, shade tree richness, and plant diversity. Using the non-metric multidimensional scaling, we tested whether there was a difference in terms of the composition of foraging guilds between habitats. Commercial polyculture coffee fields can sustain levels of bird abundance, diversity, and richness comparable to agroforestry systems under natural forest, and higher than sun coffee and shaded monoculture coffee, and of other human modified habitats such as crop/fruit fields and tree farms. Coffee agroforestry systems have a higher proportion of nectarivores, insectivores, and frugivores than other systems that can sustain high diversity and richness of birds such as paddy fields that mainly have granivores and carnivores. Complex polycultures can represent an avenue for the future of sustainable agriculture in conditions where deforestation rates are high and in crops such as coffee, which maintain high yield in the presence of diverse shade.

## 1. Introduction

Deforestation in the tropics is mainly driven by the need to expand agriculture and forestry land, resulting in around 5–10 million hectares of natural forest lost every year [1,2]. This process is not likely to lessen in the near future given the increased demand of deforestation-driven commodities [3]. A series of other indirect risks related to this expansion of agriculture and forestry land use include the spread of diseases and pests, and the increase in the trade of protected species [4,5]. Tropical cropland has further encountered a process of intensification, particularly evident in regions that are the main exporters of deforestation-driven commodities [6].

Coffee (*Coffea* spp.) is among the main commodities produced in tropical regions form Latin America, Asia, and Africa, with more than 25 million people depending on its production for their livelihoods [7]. This global commodity has a profound contribution to global biodiversity loss through agricultural extensification and intensification [4]. Nevertheless, coffee agroforestry systems have been recognised as an alternative refuge for biodiversity across different regions [8,9,10,11,12,13,14]. This is because, traditionally, coffee plants are cultivated under the forest and can maintain high yield with intermediate levels of shade cover [15,16,17]; however, there are cases when coffee productivity has decreased with increased shade cover [18]. The quality of coffee also benefits from diverse shade cover since coffee berry borer (*Hypothenemus hampei*) infestation is higher in sun-exposed fields [19]. Despite these advantages of diverse shade, coffee fields have encountered a process of agricultural intensification (i.e., reduction in crop and shade diversity and increased use of agrochemicals) during the last 30 years, allegedly to gain more revenue [20,21].

Diverse shade systems have been known to play important roles in maintaining biodiversity and crop productivity [22]. Coffee agroforestry systems are potential refugia for various wildlife including butterflies [23], birds [24,25], and mammals [16,26,27]. Evidence on the effect of shade tree removal on wildlife diversity are, however, still unclear and evidence is biased towards the Neotropics and Africa [8,15,16,18,26,27]. This is a huge gap in knowledge since the response of these interventions are taxon-specific and depend on other local factors such as biogeographical regions and resource availability.

Indonesia is the fourth largest coffee producer in the world and a global biodiversity hotspot [28,29]. Despite its importance in this industry, the impact of agriculture intensification on biodiversity is still understudied [13]. Java is the most populated island in Indonesia, with a human population density of around 1000 individuals/km^2^. The island has experienced massive deforestation since the 16th century for fulfilling global demand on agricultural production, particularly coffee production [30]. This habitat loss, combined with poaching and agriculture intensification, has reduced bird diversity, particularly in the bird species of most conservation concern [31,32]. While protected areas on Java are small and scattered [30], wildlife related exploitation for wildlife trade has a profound negative effect on avian [33,34] and mammal [35] conservation. Agroforestry systems using shade grown coffee can serve an important role in the future of biodiversity conservation on Java [13,26,33,36,37,38].

We aimed to understand the responses of birds, one of the most important taxa given their wide ecological role, between different coffee agroforestry types and other human modified habitats. In particular, we investigated the possible role of shade tree removal on bird assemblages. Given the inevitable and irreversible transition towards more intensive farming practices, understanding how species react to shade tree removal is a conservation priority. We expect that shade tree removal will have a negative effect on bird abundance, diversity, and richness, with agriculture systems with low complexity having significantly less bird abundance, diversity, and richness than agriculture systems with high complexity and under a natural forest cover. We also expect commercial polyculture coffee systems to host higher bird abundance, diversity, and richness than coffee systems with low complexity (i.e., shade monoculture coffee) or no shade (i.e., sun coffee).

## 2. Materials and Methods

### 2.1. Data Collection

**Figure 1:**
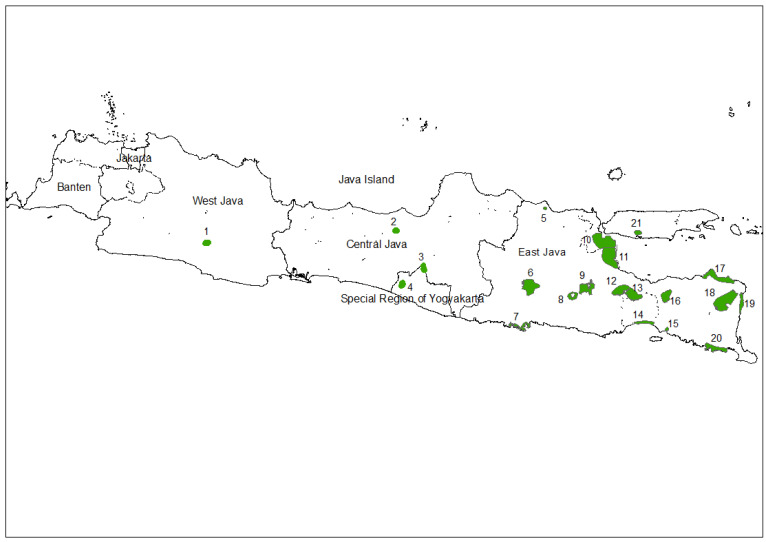
Map of 21 study areas across Java. (1) Cipaganti; (2) Kemuning; (3) Kepuharjo; (4) Jatimulyo; (5) Tuban; (6) Madiun-Kediri (7) Trenggalek; (8) Gunung Kelud; (9) Malang-Batu; (10) Surabaya-Gresik; (11) Sidoarjo; (12) Pasuruan; (13) Probolinggo; (14) Lumajang; (15) Jember; (16) Dataran Tinggi Hyang; (17) Situbondo; (18) Ijen-Baluran; (19) Banyuwangi Utara; (20) Alas-Purwo Merubetiri; (21) Sampang.

We collected data in 21 sites on Java Island from February to August 2021 (Figure 1). Java contributes around 11–13% of the total coffee production in Indonesia [39]. Coffee is produced from large plantations as well as from the agroforestry systems of small holders. We used the point sampling method for collecting data at each site with 10 min duration and 50 m radius from the observer. Three teams with extensive field experience, headed by D.A.B., F.D.R., and F.E.N. and composed of 3–4 members each, conducted the surveys between 6:00–9:00 and 15:00–18:00. In total, we collected 1128 points (Table 1). We collected data on the number of individuals for each species recorded during the sampling period. We then calculated the abundance (total abundance), diversity (Shannon Index), and richness (total number of species) in each plot. We also considered the foraging guild of each species encountered and calculated the proportion of individuals encountered for each foraging guild. In addition, we collected vegetation data through the establishment of nested plots (20 × 20 m for shade trees, 10 × 10 m for poles, 5 × 5 m for saplings, and 2 × 2 m for seedlings) in each sample point. The stages of trees were defined as follows: (i) seedling: germinated seeds to <1.5 m in height; (ii) sapling: height > 1.5 m and diameter at breast height (dbh) < 10 cm; (iii) pole: 10 cm < dbh < 20 cm; (iv) shade tree: dbh > 20 cm. We defined the habitat around each point sampling (Table S1). For the coffee agroforestry systems, we used the classification by Philpott et al. [40]. In summary, sun coffee are fields with coffee plants often mixed with other crops but no shade trees; shade monoculture coffee are fields with coffee plants often mixed with other crops and fruit trees and with shade trees but total plant richness is equal or less than five species; commercial polyculture coffee are fields with coffee plants mixed with other crops, fruit trees, and shade trees and with a total plant richness of six species or higher; traditional polyculture coffee are complex agroforestry systems under a natural forest cover; and rustic coffee systems are similar to traditional polyculture coffee systems but with a lower density of crops and a higher plant diversity. In addition, we identified the following habitats: (1) community managed forest: crops and fruit trees managed under natural forest but not including coffee plants; (2) other commercial polyculture: polyculture (i.e., agriculture field with a plant richness of more than six species) including crops, fruit trees, and shade trees but not including coffee plants; (3) other crop/fruit field: fields including crops, fruits trees, and shade trees with a plant richness of five or less species and not including coffee plants; (4) paddy field: flooded fields dominated by rice or other semiaquatic crops; and (5) tree farm: a forest managed for timber production. We also included plots dominated by mangroves in this assessment since they were in cities and often mixed with fishponds and paddy fields. We calculated the Shannon diversity index of both plant (including seedlings, saplings, poles, and shade trees) and bird species for each plot.

### 2.2. Data Analysis

To test the differences between habitats around sample points, we ran generalised additive models (GAMs) with abundance, diversity, or richness of birds per plot as response variables. We included the coordinates of the plots in the models to account for spatial autocorrelation of data via Gaussian process smooths [41]. We further included the elevation, distance to protected areas, shade tree richness, and plant diversity in the plot as other potential predictors of bird abundance, diversity, and richness [42,43,44]. We used the “gam” command in the package “mgcv” to run the GAMs. We plotted the incident rate ratios comparing habitat types with the reference category commercial polyculture coffee.

For the non-metric multidimensional scaling (NMDS), we used the proportion of observed total number of birds by foraging guilds for each site. We considered the following foraging guilds: carnivore, granivore, frugivore, insectivore, nectarivore, and omnivore. We plotted the results in terms of the main habitat in the study sites to see whether there was a difference in terms of composition of foraging guilds between habitats. We used the “metaMDS” function in the package “vegan” to run the NMDS [45]. We used R v. 4.1.0.

## 3. Results

**Figure 2:**
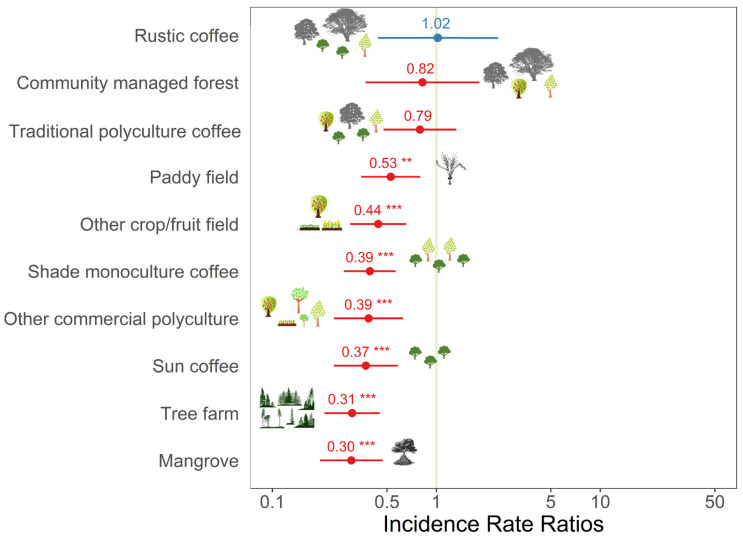
Incidence rate ratios between the abundance of birds in commercial polyculture coffee (reference category) and other habitats considered during the survey on 1228 plots in Java, Indonesia. Data are standardised beta values and 95% confidence intervals from a generalised additive model. A description of the habitats can be found in the methods. Other predictors included in the model can be found in Table 2. ** *p* < 0.01; *** *p* < 0.001.

**Figure 3:**
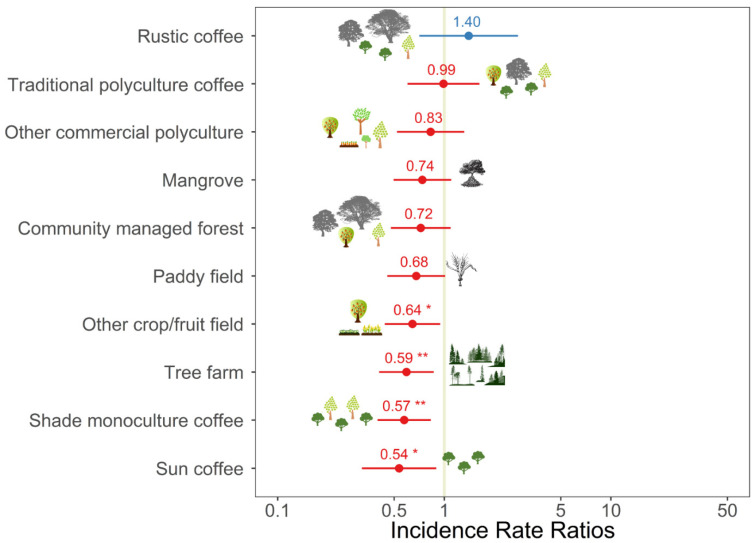
Incidence rate ratios between the diversity of birds (Shannon Index) in commercial polyculture coffee (reference category) and other habitats considered during the survey on 1228 plots in Java, Indonesia. Data are standardised beta values and 95% confidence intervals from a generalised additive model. A description of the habitats can be found in the methods. Other predictors included in the model can be found in Table 2. * *p* < 0.05; ** *p* < 0.01.

**Figure 4:**
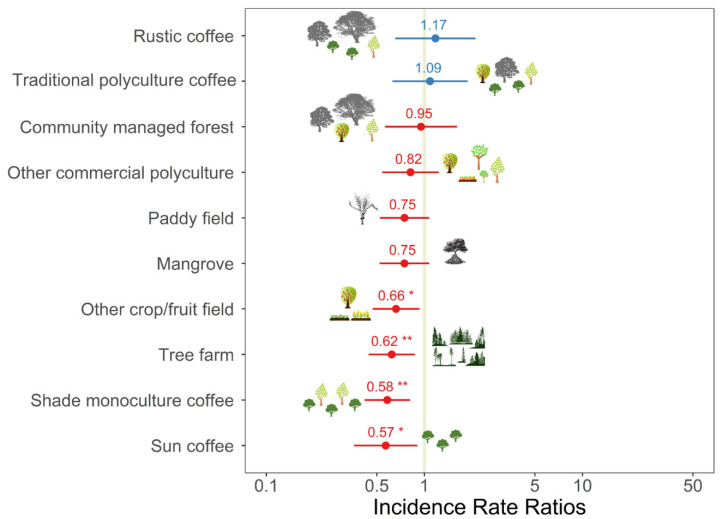
Incidence rate ratios between the richness of birds in commercial polyculture coffee (reference category) and other habitats considered during the survey on 1228 plots in Java, Indonesia. Data are standardised beta values and 95% confidence intervals from a generalised additive model. A description of the habitats can be found in the methods. Other predictors included in the model can be found in Table 2. * *p* < 0.05; ** *p* < 0.01.

The abundance of birds in commercial polyculture coffee fields (i.e., fields comprised of coffee plants, other crops and/or fruit trees, and diverse shade trees) was significantly higher than the abundance in all the other habitats apart from rustic coffee, traditional polyculture coffee, and community managed forest (Table 2; Figure 2). The diversity and richness of birds in commercial polyculture coffee fields were higher than in other crop/fruit fields, sun coffee, tree farms, and shade monoculture coffee (Table 2; Figure 3 and Figure 4). The diversity and richness of birds in commercial polyculture coffee fields were similar to the diversity/richness in traditional polyculture coffee and rustic coffee systems as well as in community managed forests. Paddy fields, mangrove patches, and other commercial polyculture fields also had a similar diversity and richness to commercial polyculture coffee.

**Figure 5:**
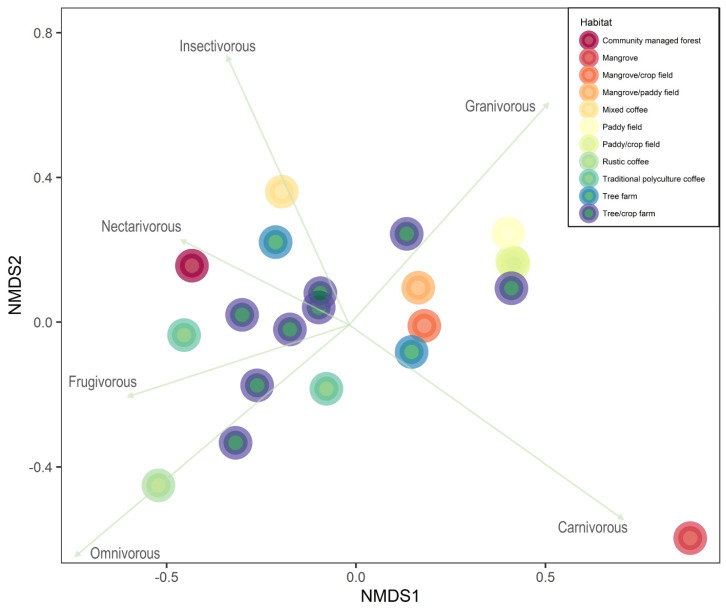
Non-metric multidimensional scaling representing the composition of foraging guilds at the 21 sites. Different colours indicate the main habitat in the study sites (or two main habitats if they were equally important).

The composition of bird communities is also different depending on the habitat. Mangrove patches mainly had carnivores, and sites mixed with mixed mangroves, paddy fields, and/or other crops had mainly carnivores and granivores. Agroforestry systems mainly dominated by coffee had a higher proportion of nectarivores and frugivores (in the case of traditional polyculture coffee), omnivores (rustic coffee), or insectivores (mixed coffee gardens ranging from sun-exposed to commercial polyculture coffee) (NMDS: stress = 0.08; Figure 5).

## 4. Discussion

Given the high rate of deforestation in the tropics, our finding that commercial polyculture coffee can sustain good levels of bird abundance, diversity, and richness that are comparable to coffee agroforestry systems under natural forest is of crucial importance. Commercial polycultures, in fact, represent the highest degree of habitat complexity that can be achieved in croplands after deforestation. Coffee polycultures hosted a higher abundance of birds than polycultures with other crops, and this is not surprising given the predisposition of coffee plants to maintain high yields and good quality coffee under a diverse shade cover [15,16,17]. We note that Indonesia offers a unique case, since, of the 1.2 million ha of coffee fields present, 96% belongs to smallholder farmers [46]. There is thus a possibility to work together with farmers and integrate wildlife-friendly practices that can be beneficial for both farmers, by producing other commodities and guaranteeing high coffee yields, and wildlife and by offering a diverse system of shade trees and crops that promote ecosystem services. Additionally, most of the commercial polyculture coffee fields considered in this study are currently included in programs where organic farming and other wildlife-friendly practices are encouraged [47].

The ecosystem services provided by coffee agroforestry systems were also increased compared to other habitats that had a similar diversity and richness of birds such as paddy fields. This is because coffee agroforestry systems have a higher proportion of insectivores, nectarivores, and frugivores, meaning that ecosystem services such as pollination, natural pest control, and seed dispersal are favoured [48]. The complexity of the agroforestry system also promotes an increase in ecosystem services, for example, the number of pollinators can also increase in coffee fields with higher complexity in terms of shade tree richness [13]. A diverse shade also provides key services such as increasing soil quality by nitrogen fixation and increasing litter biomass, protecting from direct sun, attracting pollinators, and increasing habitat connectivity [47,49].

Our study adds to the current literature that supports that coffee agroforestry systems can sustain good levels of animal diversity in Indonesia. For example, a study on Sunda leopard cat *Prionailurus bengalensis javanensis* showed that non-protected areas, particularly coffee agroforestry, outcompeted small and scattered protected areas in supporting this carnivore [33]. Another study compared the detection rates on wildlife via camera traps in a coffee agroforestry system and a protected forest and found that at least ten mammal species used the agroforestry system [50]. Only Javan leopard *Panthera pardus melas*, Sunda porcupine *Hystrix javanica*, and grizzled langur *Presbytis comata* were not detected in the agroforestry system, but the authors recognized that the matrix could bring benefits to these species by acting as a buffer zone to reduce the human pressure on the forest, reduce human–wildlife conflicts, and help maintain ecosystem services.

Our findings highlight the importance of polyculture practices that are used in various agroforestry schemes in Indonesia as alternatives in supporting wildlife conservation in the future land use change scenarios. However, such incentive mechanisms to provide biodiversity refuges in human-modified land use that can have also agricultural benefits still need to be implemented [51]. The Indonesian government promotes organic farming and provides incentives for farmers willing to convert to organic practices via the Go Organic program and the Indonesian Food Law (18/2012) [52]. The process to obtain organic certification, however, is complex and requires a long-term commitment by farmers and monetary incentives for farmers such as premium prices and payments for ecosystem services are difficult to achieve [47,53,54]. Still, promoting wildlife-friendly agroforestry systems is a promising solution to ensure the long-term sustainability of both biodiversity and the livelihoods of local farmers in Indonesia [47]. This is particularly urgent given the current predictions that there would be a production decline in Arabica coffee due to climate change and that this will result in an expansion of coffee cultivated areas of around 30% by 2050 [55]. It is important to work on persuading farmers in joining wildlife-friendly initiatives and empower farmers that wish to join such programs [47]. This solution may work better with some crops such as coffee, which allow for good yields in complex agroforestry systems and not with other crops such as oil palm, where wildlife-friendly farming is often unsuccessful due to low yields [56].

## 5. Conclusions

We presented evidence that commercial polyculture coffee can sustain similar bird abundance, diversity, and richness than traditional coffee systems under natural forest cover. We discussed the implications of this finding, suggesting a certain optimism that sustainable and complex farming systems can help deal with the likely agricultural intensification and extensification in the near future. We recognise that the implications of our findings can be applied to our context (coffee agroforestry systems in Java) and regional variations should be considered when extending our findings to other regions or crops. We suggest similar multi-site studies in other regions, especially in Southeast Asia and Africa, where coffee agroforestry systems are relatively understudied compared to the Neotropics [13,57].

## Figures and Tables

**Table 1:** Number of sampling points, elevation range, mean (±SE) plant diversity per plot, and mean (±SE) bird diversity per plot in the 21 study sites on Java.

Sites	Regency	Elevation Range (m above Sea Level)	Plant Diversity	Bird Diversity	Number of Observation Points	Main Habitat Types
Cipaganti	Garut	1300–1650	0.97 ± 0.06	0.65 ± 0.04	57	Mixed coffee systems: sun, shade monoculture, commercial polyculture
Kemuning	Temanggung	459–649	1.63 ± 0.03	1.83 ± 0.04	89	Rustic coffee
Kepuhharjo	Sleman	616–985	1.36 ± 0.07	1.34 ± 0.12	30	Traditional polyculture coffee
Jatimulyo	Kulon Progo	514–765	1.35 ± 0.07	1.10 ± 0.12	30	Traditional polyculture coffee
Ijen-Baluran	Banyuwangi	70–1595	0.20 ± 0.04	0.88 ± 0.07	46	Tree farms and other crops *
Madiun-Kediri	Kediri	780–1265	0.31 ± 0.05	1.08 ± 0.06	60	Tree farms and other crops *
Batu-Malang	Malang	881–1484	0.21 ± 0.04	0.92 ± 0.07	60	Tree farms and other crops
Pasuruan	Pasuruan	595–1793	0.50 ± 0.06	1.49 ± 0.08	60	Tree farms *
Trenggalek	Trenggalek	4–372	0.47 ± 0.06	1.19 ± 0.05	60	Tree farms and other crops *
Gunung Kelud	Kediri, Malang, Blitar	712–1031	0.29 ± 0.05	1.33 ± 0.06	60	Tree farms and other crops
Sampang	Sampang	0–8	0.22 ± 0.05	1.89 ± 0.05	60	Mangrove and paddy fields
Kota Surabaya	Surabaya	0–47	0.43 ± 0.06	1.94 ± 0.04	60	Mangrove and other crops
Probolinggo (BTS)	Probolinggo	607–1412	0.25 ± 0.06	0.99 ± 0.08	60	Tree farms
DT Hyang	Probolinggo	498–968	0.83 ± 0.06	1.14 ± 0.07	60	Community managed forest
Situbondo	Situbondo	0–32	0.11 ± 0.04	0.87 ± 0.06	60	Paddy fields and other crops
Tuban	Tuban	3–32	0.10 ± 0.04	1.49 ± 0.07	60	Paddy fields and other crops
Banyuwangi	Banyuwangi	5–182	0.44 ± 0.06	0.91 ± 0.07	60	Tree farms and other crops
Jember	Jember	1–17	0.03 ± 0.02	1.19 ± 0.06	60	Paddy fields
Lumajang	Lumajang	1–26	0.12 ± 0.05	1.12 ± 0.06	60	Tree farms and other crops
Sidoarjo	Sidoarjo	0–4	0.25 ± 0.05	2.03 ± 0.06	60	Mangrove and other crops
Alas Purwo-Meru Betiri	Banyuwangi	0–112	0.43 ± 0.06	0.86 ± 0.08	60	Tree farms

* Commercial coffee fields are present but do not represent the main habitat.

**Table 2:** Results of generalised additive models to understand the influence of habitat type and other environmental predictors on the abundance, diversity, and richness of birds in 21 sites, 1226 plots, in Java, Indonesia.

Response Variable ^a^	Predictor	Category	Estimate ± Std. Error	Z-Value	*p*	Smooth Term		*p*
						Edf	*χ* ^2^	
Bird abundance	Intercept		4.06 ± 0.40	8.24 **	<0.001			
	Habitat ^b^	Community managed forest	−0.19 ± 0.41	−0.48	0.634			
		Mangrove	−1.19 ± 0.22	−5.32 **	<0.001			
		Other commercial polyculture	−0.95 ± 0.25	−3.83 **	<0.001			
		Other crop/fruit field	−0.82 ± 0.20	−4.08 *	<0.001			
		Paddy field	−0.64 ± 0.21	−3.02 **	0.003			
		Rustic coffee	0.05 ± 0.39	0.33	0.741			
		Shade monoculture coffee	−0.93 ± 0.19	−5.02 **	<0.001			
		Sun coffee	−0.99 ± 0.23	−4.31 **	<0.001			
		Traditional polyculture coffee	−0.23 ± 0.26	−0.88	0.378			
		Tree farm	−1.18 ± 0.20	−5.94 **	<0.001			
	Shade tree richness		0.04 ± 0.02	2.03	0.043 *			
	s(plant diversity)					8.42	53.22 **	<0.001
	s(elevation)					2.58	4.54	0.177
	s(longitude, latitude)					31.68	1724.84 **	<0.001
	s(distance to protected areas)					8.07	109.00 **	<0.001
Bird diversity	Intercept		0.55 ± 0.19	2.91 *	0.004			
	Habitat ^b^	Community managed forest						
		Mangrove						
		Other commercial polyculture						
		Other crop/fruit field						
		Paddy field						
		Rustic coffee						
		Shade monoculture coffee						
		Sun coffee						
		Traditional polyculture coffee						
		Tree farm						
	Shade tree richness							
	s(plant diversity)							
	s(elevation)							
	s(longitude, latitude)							
	s(distance to protected areas)							
Bird richness	Intercept		1.75 ± 0.23	7.74 **	<0.001			
	Habitat^b^	Community managed forest	−0.05 ± 0.27	−0.19	0.853			
		Mangrove	−0.29 ± 0.19	−1.57	0.117			
		Other commercial polyculture	−0.20 ± 0.21	−0.96	0.336			
		Other crop/fruit field	−0.41 ± 0.17	−2.37 *	0.018			
		Paddy field	−0.21 ± 0.18	−1.20	0.23			
		Rustic coffee	0.11 ± 0.31	0.67	0.502			
		Shade monoculture coffee	−0.54 ± 0.17	−3.19 **	0.001			
		Sun coffee	−0.56 ± 0.24	−2.40 *	0.016			
		Traditional polyculture coffee	0.08 ± 0.28	0.29	0.769			
		Tree farm	−0.48 ± 0.17	−2.76 **	0.006			
	Shade tree richness		0.01 ± 0.02	0.84	0.387			
	s(plant diversity)					1.00	0.15	0.699
	s(elevation)					1.00	0.53	0.468
	s(longitude, latitude)					25.45	457.90 **	<0.001
	s(distance to protected areas)					2.67	3.40	0.484

^a^ fit family for bird abundance: Poisson (link = “sqrt”); bird diversity: Tweedie; bird richness: Poisson (link = “log”); ^b^ reference category: commercial polyculture coffee; * *p* < 0.05; ** *p* < 0.01.

## Data Availability

The data presented in this study are available in Table S1. Additional raw data are available on request from the corresponding author.

## Supplementary Materials

The following supporting information can be downloaded at: https://www.mdpi.com/article/10.3390/biology11020310/s1, Table S1: Dataset used for the analysis.
